# Effect of Huangqin Tang on Colonic Gene Expression in Rats with Ulcerative Colitis

**DOI:** 10.1155/2020/4238757

**Published:** 2020-03-26

**Authors:** Dunfang Wang, KaiFeng Shi, Yanli Wang, Dixin Zou, Shanshan Guo, Tao Li, Hangyu Xu, Xuran Ma, JiaXing Liu, HongXin Song, Weipeng Yang, Yu Li

**Affiliations:** ^1^Institute of Chinese Materia Medica, China Academy of Chinese Medical Sciences, Beijing 100700, China; ^2^Wang Jing Hospital, China Academy of Chinese Medical Sciences, Beijing 100102, China; ^3^The Experimental Research Center, China Academy of Chinese Medical Sciences, Beijing 100700, China; ^4^Beijing University of Chinese Medicine, Beijing 100029, China

## Abstract

In this study, we explored the pharmacological mechanisms of Huangqin Tang (HQT; a traditional Chinese medicine formula) in ulcerative colitis (UC) and provided evidence for potential roles HQT plays by gene expression profiling. The UC rat model was made via a compound method (trinitrobenzene sulfonic acid plus ethanol). After a ten-day treatment, microarray analysis was performed from the colon segment of the rats. Biological functions and specific signaling pathways were enriched based on differentially expressed genes (DEG), and corresponding gene networks were constructed via Ingenuity Pathway Analysis (IPA). Through the network, we screened the potential “candidate targets,” such as ITGB1, FN1, CASP3, and ITGA5 and FABP1, ABCB1, FABP2, and SLC51B. These potential candidate targets were functionally related to immune responses, inflammation, and metabolism. Moreover, HQT significantly decreased serum levels of proinflammatory factors nitrogen monoxide (NO), proinflammatory cytokines interleukin- (IL-) 17, and prostaglandin E2 (PGE2). The degree of HE staining of colonic tissue was severe in the model group but reduced significantly in the HQT group. HQT exhibited protective effects against colon damage by inhibiting the inflammatory response.

## 1. Introduction

UC is a chronic nonspecific inflammatory disease involving the rectum and colon, characterized by mucosal T cell dysfunction, abnormal cytokine production, and cellular inflammation that lead to damage of the distal small intestine and the colonic mucosa [1, 2]. In severe cases, visible focal hemorrhages are apparent and the colonic mucosa becomes brittle and bleeds easily [3]. The importance of mucosal healing is now acknowledged in new guidelines, which recommend incorporating complete mucosal healing (along with symptom resolution) in all therapeutic studies as the primary endpoints of remission [4]. Most current therapies for UC include corticosteroids, glucocorticosteroids, aminosalicylates, and immunosuppressive agents. However, side effects are serious and the recrudescence rates of UC are high [5, 6]. Compared with chemical treatment, herbal medicine has the advantage of less toxic side effects, which is increasingly attracting researchers' attention as a drug candidate for the treatment of such diseases [7].

HQT can be used to treat UC based on traditional Chinese medicine (TCM) theory. It has been widely used to treat gastrointestinal diseases, such as diarrhea, abdominal spasms, fever, headache, vomiting, nausea, extreme thirst, and subcardiac distention [8]. The major effective components of the four herbs are flavonoids (e.g., wogonin, baicalein, and oroxylin-A) and flavonoid glycosides (e.g., wogonoside, baicalin, and oroxylin-A-glucoside) [9–11], terpenoids, isoflavones, polysaccharides, and volatile oils. Phytochemical studies have shown that these compounds have a wide range of pharmacological characteristics [12–14], such as anti-inflammatory, antitussive, tumor suppressor, analgesic, and immunomodulatory properties [15–18]. Flavonoids from *Radix Scutellariae* are the major active ingredients from HQT.

With the soaring development of systems biology and polypharmacology, “network pharmacology” [19, 20] has shifted the paradigm of “one gene, one drug, one disease” to the “multi-component, network target” strategy [21], of which the key idea is in line with the holistic theory of TCM. To explore the pharmacological mechanisms of HQT in UC, we used genome-wide microarray detection and ingenuity pathway analysis (IPA) to identify 569 various genes (199 upregulated and 370 downregulated genes, ∣fold change (FC)∣ ≥ 1.5, *p* value < 0.05) in the model group compared to the normal group. Further, after data processing of the 569 genes, we found 162 genes (∣fold change (FC)∣ ≥ 1.3, *p* value < 0.05) in the HQT group compared to the UC model group. We were then able to construct the network using the 162 the potential regulating genes of HQT by Cytoscape. Among them, we analyzed potential “candidate targets” (ITGB1, FN1, CASP3, and ITGA5 and FABP1, ABCB1, FABP2, and SLC51B) in terms of the “degree” ≥10 or |fold change (FC)∣ ≥ 4 in the network. These potential candidate targets were functionally correlated with proliferation, inflammatory response, and metabolism, and these genes were revealed to be crucial components in the pathways of inflammation. Moreover, portions of gene expression values were validated by qRT-PCR. All in all, the integrative research combining microarray gene expression profiling, network analysis, and inflammation experiment reveals convincing evidence that HQT may alleviate UC via regulating immune or metabolism-related genes in a holistic way.

## 2. Materials and Methods

### 2.1. Animals

Male Wistar rats were obtained from the Laboratory Animal Center of the Academy of Military Medical Sciences; Production license No. was SCXK 2012–0004, weight 180–200 g. All rats were housed at 23 ± 1.5°C. Animal experiment process was conducted in accordance with the ethical guidelines for local animal care and usage.

### 2.2. Preparations of HQT

*Scutellaria baicalensis Georgi*, *Paeonia lactifloraPall*, *Glycyrrhiza uralensis Fisch*, and *Ziziphus jujuba Mill* (weight ratio 3 : 2 : 2 : 3) were weighed and mixed. For the first decoction, the mixture was refluxed with water (1 : 10, *w*/*v*) for 1.5 h. The filtrates were collected; then, the residues in water (1 : 8, *w*/*v*) were boiled for an additional 1 h. Two batches of filtrates were gathered. Hereafter, the sample is dried under reduced pressure to obtain HQT extract. In addition, the major active chemical compounds of each herb containing in HQT were determined by liquid chromatography–tandem mass spectrometer (LC-MS).

### 2.3. UC Rat Model Construction and Grouping

TNBS colitis in rats was induced according to previously reported methods [22]. 24 h fasted Wistar rats were moderately anesthetized with pentobarbital then carefully inserted a 0.56 mm catheter into the colon with the tip 8 cm proximal to the anus. To break the intestinal epithelial barrier and induce colitis, the mixed reagent containing 0.25 ml of 50% ethanol and TNBS (100 mg/kg) was slowly administered into the lumen by the catheter fitted onto a 1 ml syringe. Rats in the control group were all administered an equal volume of saline. Animals were kept in an upside-down position for 2 min and returned to their cages.

For microarray analysis, male rats were divided into three groups randomly: a normal control group, a UC model group, and a group in which UC rats were treated with HQT formula (HQT group). Each group has 10 rats. The HQT group was administered orally at a daily dose of 20 g/kg for 10 consecutive days. Rats in the control and UC model groups received an equal volume of saline.

### 2.4. Gene Expression Data Analysis

On the 10th day, rats were killed under anesthesia. The colonic segments were collected from all three rat groups, freed of adherent adipose tissue, and washed with cool saline. Colonic segments were then frozen in liquid nitrogen immediately for further microarray detection. Whole transcriptome analysis was developed by Affymetrix GeneChip®Rat Gene 2.0 Array chips. Gene array data was uploaded to the IPA system. A cutoff was set to identify genes whose expression was significantly differentially regulated; these molecules were known as Network Eligible Molecules (NEMs). Networks of NEMs were then algorithmically formed based on their connectivity. The probability of the assignment was expressed by a *p* value calculated using the right-tailed Fisher's exact test. The level of statistical significance was set at *p* < 0.05. Subsequently, signal pathways with a *p* value < 0.05 and ∣fold change (FC)∣ ≥ 1.5 were screened out and analyzed [23–28].

### 2.5. Serum Cytokine Detection

After a ten-day treatment, blood samples were collected by eyelid method and then centrifuged (3,000 rpm, 15 min) to get serum. Production of NO in serum was measured by Griess assay, and the levels of proinflammatory cytokines IL-17 and PGE_2_ in serum were detected by ELISA kits according to the manufacturer's specifications.

### 2.6. Histological Examination

After tissue collection, the tissue was embedded and sliced as usual. Then, 4 *μ*m-thick tissue sections were prepared and stained with hematoxylin-eosin (HE) for histological studies. The extent of colonic lesions was compared based on ulcer size, inflammatory infiltration, and structural damage.

### 2.7. Reverse Transcription-PCR

SYBR Green 1 kit was applied under the manufacturer's instructions, and the control housekeeping gene GAPDH was utilized as a reference gene. Then, 2 *μ*g of total RNA was performed for reverse transcription using ABI High-Capacity cDNA reverse transcription Kits. Primer sequences of target genes are given in Table 1. Moreover, the mixtures were diluted tenfold and final reaction volumes of 20 *μ*l were used for data analysis using BIO-RAD CFX Manager Version 3.0 software. Reactions were run in triplicate. The relative levels of target genes were calculated by the 2^−ΔΔCt^ method [29].

### 2.8. Statistical Analysis

SPSS version 11.0 (SPSS Inc., Chicago, IL, USA) was used for statistical analysis. Values displayed represent the mean ± standard deviation (SD). Differences between mean values were analyzed by one-way analysis of ANOVA followed by LSD method. Significance of differences were accepted at *p* values less than 0.05.

## 3. Results

### 3.1. Chemical Compounds of HQT

LC-MS was performed to identify the chemical compounds of each herb contained in HQT. The 10 main active ingredient chemical compositions were as follows: Baicalin (10.4149%), Wogonoside (2.4179%), Oroxylin-A-glucoside (0.8229%), Baicalein (0.6754%), Wogonin (0.2215%), Oroxylin-A (0.0967%), Liquiritin (0.0384%), Isoliquiritin apioside (0.0354%), Liquiritigenin (0.0280%), Isoliquiritoside (0.0865 *μ*g/mL), and Isoliquiritigein (0.0049%) [30].

### 3.2. HQT Promoted Recovery of the UC Rats

The UC rats induced by TNBS showed a series of symptoms within 3 days, such as apathetic, drowsiness, dull hair, poor appetite, weight loss, diarrhea, and purulent stools. After prolonged HQT exposure, rats_,_ response to above symptoms were all improved to varying degrees. Their appetite was stimulated with decreased diarrhea. There was no significant improvement in symptoms in the model group rats.

### 3.3. HQT Decreases Serum Level of Inflammatory Cytokines

The levels of proinflammatory factors NO, IL-17, and PGE2 in the model group were increased remarkably in model group. In addition, levels of NO, IL-17, and PGE2 in the HQT group were clearly lower than those in the model group (Table 2).

### 3.4. Histological Study

**Figure 1:**
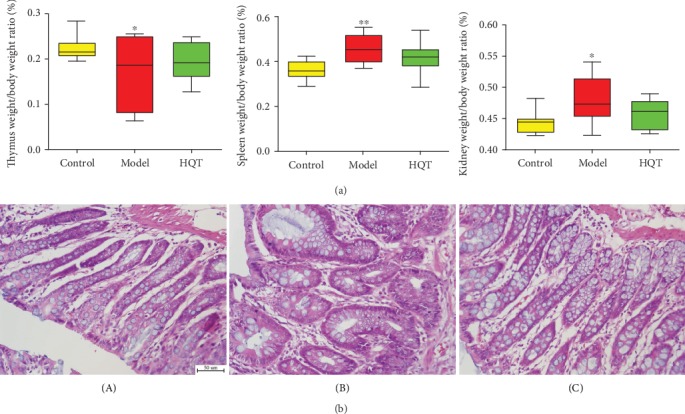
Effect of HQT on TNBS-induced ulcerative colitis rats. (a) Weights of the spleen and kidney were, respectively, increased in the UC model group compared to the normal group; after treatment with HQT, the two parameters were both lower than those in UC model rats. Weight of the thymus was reduced in the UC model group compared to the normal group; after treatment with HQT, the indicator was higher than that in UC model rats; (b) histological observations of the colon tissues in different groups (HE staining), (10 × 40). A: normal control; B: UC model control; C: HQT 20 g·kg^−1^. Data are represented as the mean ± SE. ^∗^*p* < 0.05 vs. normal control.

The rats with TNBS-induced colitis in the mucosa of colons revealed inflammatory cell infiltration, loss or enlarged of goblet cells and epithelium, and edema. Part of the colon gland disappeared. The histologic sections of the HQT treatment group showed progressive restoration, improvement of intestinal ulcer, and reduction in infiltration of inflammatory cells and edema compared to that of the TNBS control group (Figure 1(b)). Considering internal organs as the executors of the physiological function of the body, the organ coefficient can be approximated to reflect the functional state of organs and disease. In this experiment, we observed that the weight of the spleen and kidney were, respectively, increased (*p* < 0.05, *p* < 0.01) in the UC model group compared to the normal group; after treatment with HQT, the two parameters were both lower than those in UC model rats (*p* > 0.05). While the weight of the thymus was reduced (*p* < 0.01) in the UC model group compared to the normal group, after the administration of HQT, the indicator was higher than those in UC model rats with no statistical significance (Figure 1(a)).

### 3.5. Heat Maps of Relative Expression Changes

**Figure 2:**
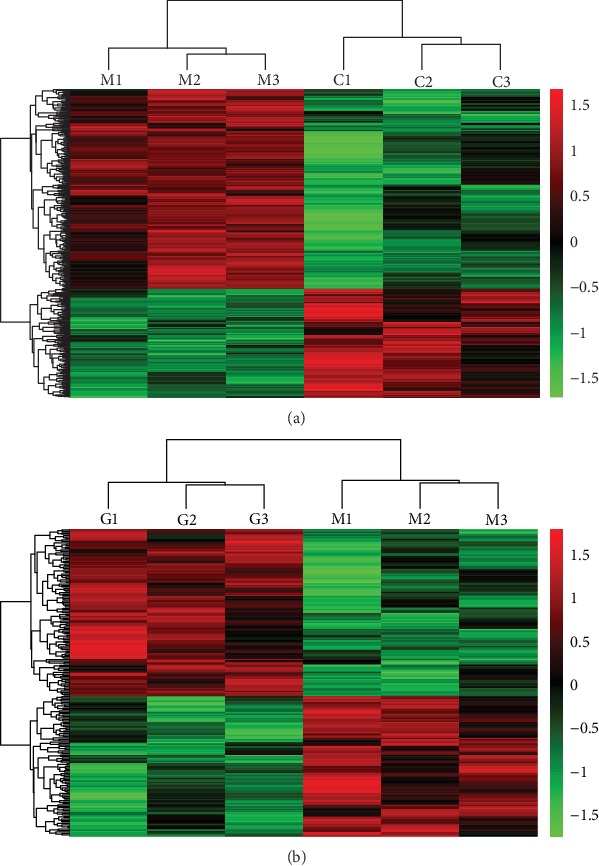
(a) Unsupervised hierarchical clustering analysis of all dysregulated genes in UC model and normal control rats and (b) of all dysregulated genes in HQT and UC model groups. Red represents a high expression while green represents a low expression, and black represents no difference. M1~M3: three samples in UC model group; C1~C3: three samples in normal control group; G1~G3: three samples in HQT group.

By data processing and DEG screening, there were 199 upregulated and 370 downregulated genes in UC model rats compared to the normal rats. After HQT-treated, there were 114 upregulated and 90 downregulated genes in HQT rats compared to the UC model rats. In addition, unsupervised hierarchical clustering analysis (Figure 2(a)) of the dysregulated genes presented a good differentiation of normal and model samples, suggesting the successful construction of the UC model induced by TNBS. After HQT treatment, dysregulated genes tended to callback (Figure 2(b)).

### 3.6. HQT Alleviates UC Via Regulating Potential Key Genes

**Figure 3:**
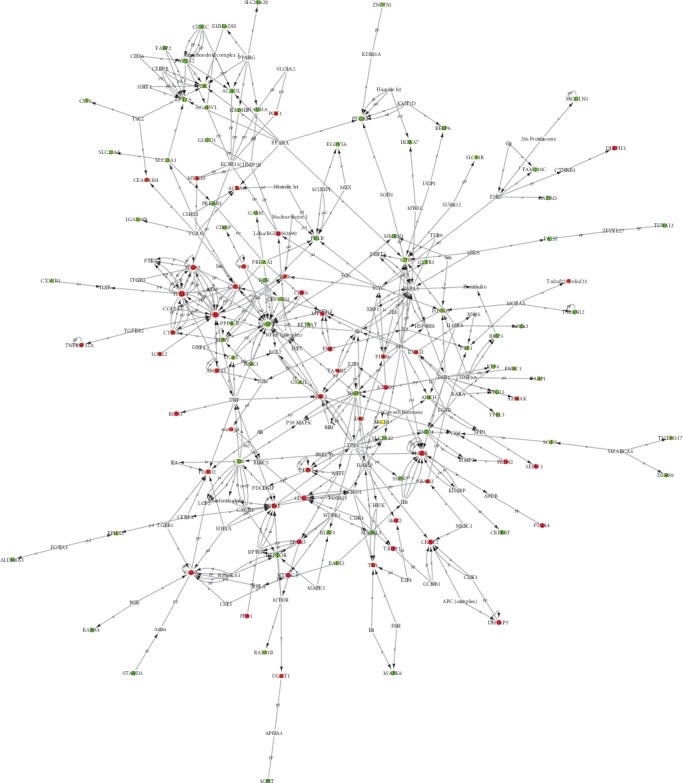
Analyses of interactions among 162 genes. Red and green nodes represent the differentially expressed genes identified in these networks. A red node denotes an upregulated gene, a green node denotes a downregulated gene, and a yellow node denotes a related inflammation gene. Genes in gray notes were not identified as differentially expressed in our experiment and were integrated into the computationally generated networks based on the evidence stored in the IPA database, indicating relevance to these networks.

According to the IPA analysis, 162 genes (genes with a *p* value of <0.05 and a ∣fold change (FC)∣ ≥ 1.5, but with a ∣fold change (FC)∣ ≥ 1.3 after being HQT-treated) were viewed as significantly differentially expressed between the model and HQT groups. We then constructed the network utilizing interaction information among HQT-regulated genes (Figure 3). According to the pathway enrichment analysis and top diseases and functions based on IPA, the potential “candidate targets” (including ITGB1, FN1, and ITGA5) of the network were frequently involved Actin Cytoskeleton Signaling, Agranulocyte Adhesion and Diapedes, Integrin Signaling, NF-*κ*B Activation by Viruses, FAK Signaling, PAK Signaling, and ERK/MAPK Signaling.

### 3.7. Top Diseases and Functions

Based on potential gene interactions among the HQT-regulated genes, we applied the “Core” program of the IPA Software to identify biological and functional networks. The majority of these genes were classified into ten networks comprising functions (Table 3).

### 3.8. Experimental Verification of mRNA Levels

**Figure 4:**
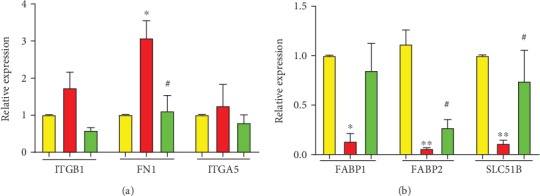
Effect of HQT on mRNA expression levels of the corresponding genes according to real-time PCR analysis. Data are represented as the mean ± SE.^∗^*p* < 0.05 and ^∗∗^*p* < 0.01 in comparison with the normal control group. ^#^*p* < 0.05 and ^##^*p* < 0.01 compared with the model group.

Real-time PCR experimental verification showed that the therapeutic effects of HQT on UC rats were consistent with findings based on microarray dates (Figure 4). Moreover, the results of real-time PCR analyses indicated that the mRNA levels of FN1 (*p* < 0.05), FABP1 (*p* < 0.05), FABP2 (*p* < 0.01), and SLC51B (*p* < 0.01) were up- or downregulated with statistical significance while ITGB1 and ITGA5 were upregulated with no statistical significance. FABP1, FABP2, and SLC51B expressions in the colon tissues of UC rats were lower than those in normal rats, but could be effectively increased by HQT treatment, and the increased FN1 expression in UC model rats markedly reduced in the HQT group.

## 4. Discussion

From a systematic perspective and at a molecular level, we combined genome-wide microarray detection based on the colon tissues of UC rats and network target analysis to define HQT in the current study. Notably, a list of differentially expressed genes screened based on fold change and degrees in the interaction network were identified including FABP1, ABCB1, FABP2, SLC51B, ITGB1, FN, and ITGA5 (Tables 4 and 5). According to the top diseases and functions, these potential candidate targets were most significantly related to cellular growth and proliferation, cellular recombination, repair, and metabolism, all of which appear to be involved in UC progression. Moreover, the potential regulating genes were also found as crucial components in some pathways. These genes drew our attention, and we further analyzed them to explore pharmacological mechanisms of HQT acting in the setting of UC.

Integrins including ITGB1 are membrane receptors involved in cell adhesion and several processes, including immune response. ITGB1 is expressed during hypoxic conditions and can serve as an indicator of intestinal wound repair [31]. The increased levels of ITGB1 observed in the inflamed colon are consistent with previous studies that reported murine ITGB1 to be induced in a TNBS concentration-dependent manner [31]. Considering the ITGB1 gene, which functions in regulating cell adhesion, we analyzed HQT-mediated modulation of cell adhesion-related genes that may affect the attachment of one cell to another cell, suppress inflammation, and play a critical role in intestinal epithelium protection.

FN1, related to migration processes and cell adhesion, is poorly expressed in normal adult tissue but overexpressed in wound healing [32]. At the same time, our study here found FN1 to be elevated in the colon of the UC rat and HQT to effectively inhibit the expression of FN1. As another member of the integrin family, ITGA5 can combine with ITGB1 and form a heterodimer “Integrin *α*5*β*1.” Integrin *α*5*β*1 is a fibronectin (FN) receptor. Signal transduction could be triggered when Integrin *α*5*β*1 combined with FN1 to activate a variety of signaling molecules and participated in physiological processes such as cell adhesion and cell skeleton reconstruction [33]. According to canonical pathways, the genes ITGB1, ITGA5, and FN1 all associate with the pathways, such as agranulocyte adhesion and diapedes, NF-*κ*B activation by viruses, FAK signaling, PAK signaling, and ERK/MAPK signaling. Activation of the ERK/MAPK and NF-*κ*B pathways could trigger the expression of a series of proinflammatory factors such as IL-1*β*, NO, and PGE2. Destruction of the body's immune homeostasis ultimately leads to UC [34, 35]. Our previous studies have shown that HQT can effectively inhibit the activation of ERK/MAPK and NF-*κ*B pathways, thereby downregulating inflammatory mediators [36]. Experiments also show that HQT inhibits high expression of IL-17, NO, and PGE2. It is suggested that ITGB1, ITGA5, and FN1 may be involved in the regulation of inflammation by ERK/MAPK and NF-*κ*B pathways.

Intestinal-type fatty acid-binding protein (I-FABP) has been used as a marker for the detection of acute intestinal injury [37]. The transport of fatty acids is believed to be performed by the FABP family. The result showed that FABP1 and FABP2 were downregulated in the inflamed UC mucosa. Our previous studies have shown that there was a large accumulation of fatty acids in UC rats [38]. The large accumulation of fatty acids can exacerbate the inflammatory response. The expression of FABP1 and FABP2b was upregulated in the HQT group. We raise the possibility that HQT enhanced the fatty acid transport and thus reduced the inflammatory response in UC rats.

Expression of SLC51B, which has a role in bile acid transport, was decreased in the UC group. The diminished uptake of bile acids into ileocytes is most likely responsible for the decreased expression of SlC51B in the ileum [39]. HQT can effectively increase SLC51B and FABP family gene expression, and we assume that the cells exist as a whole in response to disturbances. This leads to the activation of a range of pathways including functions such as metabolic regulation.

It is known that ABCB1 gene product P-glycoprotein (P-gp) exerts its barrier function by means of an efflux transport of drugs [40]. As ABCB1 mediates the efflux transport of xenobiotics and bacterial toxins, its downregulation in inflamed UC intestinal regions may be associated with compromised barrier function.

Analyzing the combination of organ coefficient, the thymus, as a central control organ of the immune system was decreased, and coefficients of the kidney and spleen, which are closely associated with energy metabolism, were increased in the UC model group as compared to the normal group. A previous study [41] suggests that persistence of disordered lipid metabolism can lead to kidney damage. We suspect that the changes of organ coefficients may be linked to metabolic disorder. After administration of HQT, compared to the UC model group, organ coefficients and the genes with the function of metabolism or transport of the HQT group rendered a callback trend, indicating that HQT could regulate dysfunctions of lipid and fatty acid metabolism, further improving the state of organ function.

In summary, this integrative study offers convincing evidence that HQT may alleviate UC via regulating the aforementioned potential key genes, which are involved in immune responses, inflammation, and metabolism. These novel findings may provide a novel and powerful mean to clarify HQT as an efficient drug candidate in therapy of UC.

## Figures and Tables

**Table 1:** Primers used for the quantitative PCR.

Gene	Primer sequence 5′ → 3′
ITGB1	Forward: 5′-GAACTTGTTGGTCAGCAGCG-3′
	Reverse: 5′-CAGGTGACACTGGCCATCAT-3′
ITGA5	Forward: 5′-CAAGGTGACGGGACTCAACA-3′
	Reverse: 5′-AACACTTGGCTTCAGGGCAT-3′
FABP1	Forward: 5′-TACCAAGTGCAGAGCCAAGAG-3′
	Reverse: 5′-TGACCTTTTCCCCAGTCATGG-3′
FABP2	Forward: 5′-TGGGCATTAACGTGGTGAAGA-3′
	Reverse: 5′-GTCCAGGTCCCAGTGAGTTC-3′
SLC51B	Forward: 5′-TGGTGATGGTGATAGGCGTG-3′
	Reverse: 5′-GCGTCTCTCTTAGGATGCCC-3′
FN1	Forward: 5′-CTCATCAGTTGGGAACCCCC-3′
	Reverse: 5′-GATGGAAACTGGCTTGCTGC-3′
GAPDH	Forward: 5′-GAGTCAACGGATTTGGTCGT-3′
	Reverse: 5′-GACAAGCTTCCCGTTCTCAG-3′

**Table 2:** Effects of Huangqin Tang (HQT) on concentrations of NO, IL-17, and PGE_2_ after 10 days of oral administration on ulcerative colitis rats.

Group	Dose/g·kg^−1^	NO/*μ*mol·L^−1^	IL-17/ng·L^−1^	PGE_2_/ng·L^−1^
Normal control	—	3.36 ± 0.61	11.98 ± 1.81	388.30 ± 46.20
Model control	—	4.17 ± 0.75^∗^	14.51 ± 2.46^∗^	441.31 ± 57.49^∗^
HQT	20	3.59 ± 0.39^**#**^	12.41 ± 1.37^**#**^	397.71 ± 24.28^**#**^

(*n* = 10, ^−^*x* ± *s*) ^∗^*p* < 0.05, ^∗∗^*p* < 0.01 vs. normal control; ^#^*p* < 0.05, ^##^*p* < 0.01 vs. model control.

**Table 3:** Specific genes classified based on diseases and function.

Gene network	Top diseases and functions	Genes involved	Number of genes
1	Energy production, lipid metabolism, small molecule biochemistry	ACADL, ACADVL, CAT, CIDEC, CPT1A, EHHADH, FABP1, FABP2, GALM, HADHA, HADHB, mir-154, MT-CO1, PDK4, PKLR, PNPLA2, PRKAA1, PRKAB1, SLC22A1, SLC22A5, Tnfrsf22/Tnfrsf23, VASP	22
2	Cellular development, cellular growth and proliferation, embryonic development	ABCB1, ACTA1, AHNAK, ATAD2, BAG2, CASP3, CD4, CIRBP, CST6, DES, DHRS9, DIAPH3, EDN1, GLUD1, MMRN1, PINK1, PPA1, TNFRSF12A	18
3	Cellular movement, cellular assembly and organization, connective tissue development and function	ALOX15, BMP3, BMP4, BOK, CTGF, CYR61, ENAH, FBLN2, ITGA5, LOX, PLOD2, PRF1, PRR7, SSPN	14
4	Amino acid metabolism, small molecule biochemistry, cell morphology	CBS/CBSL, CTH, ITGB1, Ldha/RGD1562690, LDLR, PADI2, PDIA4, PDIA6, RAB9A, RRM2, SLC2A12, SOD3, STARD3	13
5	Cellular assembly and organization, DNA replication, recombination, and repair, cell cycle	CDC6, CKAP2, DEPTOR, DLGAP5, EIF4E, FAM110C, LOXL2, MAPK6, MCOLN1, PNRC1, RETSAT, TK1, YPEL3	13
6	Neurological disease, organismal injury and abnormalities, cancer	ADGRL3, ALDH6A1, CEP55, CX3CR1, GLYR1, MPST, NT5DC3, RCN3, SPAG5, Sult1a1, TRIP13, UGGT1, ZNF24	13
7	Cancer, cell cycle, cell death, and survival	Ankh, ATG13, BLZF1, CADM3, CDCA8, CEACAM4, KIF20A, OXA1L, SEPW1, SLC25A20, SLC2A12, TUBAL3	12
8	Lipid metabolism, molecular transport, small molecule biochemistry	EPHX1, FA2H, RAB11B, REEP6, RTP4, SLC51B, TAS1R2, TSPAN12, ZNF750	9
9	Cellular function and maintenance, connective tissue development and function, skeletal and muscular system development and function	CREBRF, FN1, HOXA7, MYH10, PPP1CB, PTGR1, SMC2, TMEM117	8
10	Lipid metabolism, nucleic acid metabolism, small molecule biochemistry	ELOVL6, HPGDS, LGALS9B, PGK1, WT1	5

**Table 4:** List of HQT-regulated genes screened in terms of the “degree”.

Gene name	Description	Location	Style	Degree	Biological process
ITGB1	Integrin, beta 1	Plasma membrane	Up	25	G1/S transition of mitotic cell cycle
FN1	Fibronectin 1	Extracellular space	Up	23	Ossification
ITGA5	Integrin, alpha 5 (fibronectin receptor, alpha polypeptide)	Plasma membrane	Up	15	Cell-substrate junction assembly
CD4	Cd4 molecule	Plasma membrane	Down	13	Cytokine production
VASP	Vasodilator-stimulated phosphoprotein	Plasma membrane	Up	13	Neural tube closure
CTH	Cystathionase (cystathionine gamma-lyase)	Cytoplasm	Down	11	Glutathione metabolic process
RRM2	Ribonucleotide reductase M2	Nucleus	Up	11	Mitotic cell cycle
CTGF	Connective tissue growth factor	Extracellular space	Up	10	

Note: “degree” represents the interaction information between genes. In general, the larger values suggested more associations.

**Table 5:** List of HQT-regulated genes screened in terms of “fold change” (FC).

Gene name	Description	Location	Family	FC	Biological process
FABP1	Fatty acid-binding protein 1	Cytoplasm	Transporter	-20.47	Transport
ABCB1	ATP-binding cassette subfamily B member 1	Plasma membrane	Transporter	-11.77	G2/M transition of mitotic cell cycle
FABP2	Fatty acid-binding protein 2	Cytoplasm	Transporter	-8.28	Fatty acid metabolic process
SLC51B	Solute carrier family 51 beta subunit	Plasma membrane	Transporter	-6.86	Transport
FA2H	Fatty acid 2-hydroxylase	Cytoplasm	Enzyme	-4.7	Sebaceous gland cell differentiation
CIDEC	Cell death-inducing DFFA-like effector c	Cytoplasm	Other	-4.58	Transcription, DNA-templated

## Data Availability

The datasets used and analyzed during the current study are available from the corresponding author upon reasonable request.
